# DOES COVID-19 PANDEMIC AFFECT THE ELIMINATION OF TUBERCULOSIS? LESSONS LEARNED FROM INDONESIA

**DOI:** 10.21010/Ajidv19i2.1

**Published:** 2025-04-07

**Authors:** UTOMO Budi, FATMANINGRUM Widati, SULISTIAWATI Sulistiawati, KHUEN Chan Chow, FAUZIYAH Shifa, ADNYANA I Made Dwi Mertha, ELJATIN Dwinka Syafira, SUCIPTO Teguh Hari

**Affiliations:** 1 Department of Public Health and Preventive Medicine, Faculty of Medicine, Universitas Airlangga, Tambaksari, 60132, Surabaya, East Java, Indonesia; 2 Department of Biomedical Engineering, Faculty of Engineering, University of Malaya, Malaysia; 3 Doctoral Program of Medical Science, Faculty of Medicine, Universitas Airlangga, Tambaksari, 60132, Surabaya, East Java, Indonesia; 4 Universitas Hindu Indonesia, Bali, Indonesia; 5 Faculty of Health and Medicine, Institut Teknologi Sepuluh Nopember, Surabaya, East Java, Indonesia; 6 Institute of Tropical Disease, Universitas Airlangga, Surabaya, East Java, Indonesia

**Keywords:** Tuberculosis, tropical disease, infectious disease, East Java

## Abstract

**Background:**

Tuberculosis is one of the tropical diseases which still exist in the tropical country specifically Indonesia. This study aims to investigate the recent epidemiology of tuberculosis before and during the pandemic of COVID-19.

**Materials and Methods:**

Data were collected from East Java Health Profile 2019 and 2020 which was provided by East Java Health Officer. Data were extracted and analyzed by statistical software SPSS and QGIS Application for the geographical map interpretation.

**Results:**

This study shows that the total cases of tuberculosis in 2019 was 606,985 cases, while in 2020 was decreased to 384,752 cases. The incidence rate of tuberculosis in 2019 was 224.98 per 100,000 populations, while in 2020 was 134.58 per 100,000 populations. The province with the highest amount of tuberculosis in 2019 was West Java with 143,935 cases, and also in 2020 with 90,905 cases. The province with the lowest number of tuberculosis was North Kalimantan with 2,113 cases, while in 2020 were 985 cases. The highest incidence rate of tuberculosis in 2019 was found in Jakarta which was 443,47 per 100,000 population, while in 2020 was found in Papua which was 279.92 per 100,000 population. The lowest incidence rate in 2019 was found in Bali Province which was 102.72 per 100,000 population, while in 2020 was 67.43 per 100,000 population.

**Conclusion:**

There was a decrease in TB cases before the COVID-19 pandemic (2019) compared to during the COVID-19 pandemic (2020).

## Introduction

On December 31, 2020, China first reported a cluster of cases of atypical pneumonia caused by SARS-CoV-2 (Lu *et al.*, 2020). More than 68.5 million people were infected by the virus, and more than 1.5 million had died as a result of SARS-CoV-2 (WHO, 2020). COVID-19 pandemic resulted in a multidimensional impact on healthcare globally, among them is the direct effect on the treatment of chronic diseases and infectious diseases such as tuberculosis (Nikolayevskyy *et al.*, 2021). Tuberculosis (TB) is an infectious disease that is associated with mortality and morbidity globally. In 2019, it was estimated that there will be 10 million new TB cases and 1.5 million deaths caused by TB (WHO, 2020). Over the past few decades, efforts to suppress the TB epidemic have gradually increased. By the end of 2019, seventy-eight countries (including seven High Burden Countries (HBC)) were on the track to eliminate TB cases in 2020 by reducing TB cases rate by 80% and TB deaths rate by 90%.

According to a Global Fund report from 502 health facilities in Asia and Africa, the number of TB referrals decreased by 59% in the second and third quarters of 2020 compared to the same period in 2019 (WHO, 2020; The Global Fund, 2021). Data from the World Health Organization (WHO) in March 2021 shows that COVID-19 has severely impacted more than 84 countries with an estimated 1.4 million people having undergone TB treatment in 2020 (WHO, 2021). Infected patient with TB and COVID-19 experienced poor treatment outcomes. Strict isolation conditions at home for a long period will be an extra risk factor for spreading TB bacilli to household members, especially in low socioeconomic conditions and dense populations, which is a major risk factor for TB (Alene *et al.*, 2020). At the start of the COVID-19 pandemic i.e., in early 2020, findings from the TB partnership estimated a global decline in TB case detections of around 25% from April to June 2020, compared to detection rates before COVID-19 in 2019. This variation also estimates the occurrence of additional 6.3 million TB cases and an additional 1.4 million TB deaths caused by COVID-19 between 2020 and 2025 (Cilloni *et al.*, 2020; Hogan *et al.*, 2020). According to one study model, if the COVID-19 pandemic triggers a 3-month decline in TB detection rates, the prediction is that there will be a 13% increase in TB-related deaths; including Indonesia, which experienced a decline in the discovery of new TB cases by 25% to 30% (Cilloni *et al.*, 2020).

Indonesia is the second country with the highest annual incidence of TB and has become a national problem in the last decade. The annual number of new TB cases increased to 25.40 per 1 million of the population with a treatment success rate of 88%. East Java, Indonesia is the province that occupies the second position in Indonesia in terms of TB cases. The TB situation in East Java is still not encouraging. The East Java Provincial Government continues to make maximum efforts in early detection and management of TB cases, by resolving various obstacles and challenges including technical aspects of handling cases and socio-technical aspects of management. Also the socio-economic culture of the community is factored into the solution of the problems. In addition, the current state of the COVID-19 pandemic can worsen TB eradication efforts in Indonesia. The emergence of the current pandemic, especially in Indonesia, is a new challenge in controlling infectious diseases. As an infectious disease, COVID-19 is caused by the highly contagious SARS-CoV-2 virus that can be transmitted through droplets, either directly or indirectly (Handayani *et al.*, 2021; WHO, 2019). Data presentation and information from various surveys or reports will be meaningless if there is no elaboration and constructive use. Therefore, more epidemiological study will be needed to show the magnitude of TB cases before and after the COVID-19 pandemic, especially in the province of East Java, Indonesia (Handayani *et al.*, 2021; Caren *et al.*, 2022).

## Material and Methods

### Study Area

Indonesia is located between 6^o^ North Latitude to 11^o^ South Latitude and 95^o^ to 141^o^ East Longitude, between Australia and Asia continents, and between the Indian and the Pacific Oceans. Indonesia is also an archipelago country with 17,504 islands. Indonesia consists of 34 provinces, 7,230 districts, 8,488 sub-districts, and 74,953 villages.

### Data Collection

This is a retrospective study. Data were collected from the Indonesian Ministry of Health website which provides annual epidemiological data. Data were obtained from https://pusdatin.kemkes.go.id/resources/download/pusdatin/profil-kesehatan-indonesia/Profil-Kesehatan-Indonesia-Tahun-2020.pdf which was available to the public.

## Results

A two-year period of national data on TB was analyzed. The data consisted of the regional report from 34 provinces across Indonesia.

a. The number of TB cases

This study revealed that in 2020, the total number of TB cases was 351,936 cases, in comparison to 2019 which was 543,874 cases. Case notification rate/100,000 populations were also lower than in 2019. Interestingly, the treatment coverage in 2020 was 44%, which was higher than in 2019 which stood at 38.5%.

**Table 1:** Comparison of TB cases among children between 2019 and 2020.

No	The number of TB case	2019 (%)	2020 (%)
1	Male	313.642 (57,66%)	203.243 (57,74%)
2	Female	230.232 (42,34%)	148.693 (42,25%)
3	Male and Female	543.874	351.936
4	TB cases in Children	63.111 (11,6%)	32.816 (9,32%)
5	CNR/100 K Populations	204	125
6	Treatment Coverage %	38,5	44

CNR: Case notification rate

**Table 2:** Age distribution of TB cases in 2019 and 2020.

Age groups	No. (%) of TB cases 2019	No. (%) of TB cases 2020
0-14	64.840 (11,96%)	25.922 (7,81%)
15-24	84.543 (15,59%)	56.784 (17,11%)
25-34	86.437 (15,94%)	59.004 (17,78%)
35-44	84.133 (15,52%)	49.230 (14,83%)
45-54	89.597 (16,53%)	60.785 (18,3%)
55-64	78.347 (14,45%)	49.300 (14,85%)
>65	54.053 (9,97%)	30.805 (9,28%)

**Table 3:** Age distribution of confirmed culture-positive TB cases in 2019 and 2020.

Age groups	No. (%) of TB cases 2019	No. (%) of TB cases 2020
0-14	4.302 (1,65%)	2.373 (1,46%)
15-24	42.811(16,44%)	28.109 (17,38%)
25-34	46.210 (17,75%)	30.439 (18,82%)
35-44	47.716 (18,33%)	29.272 (18,1%)
45-54	51.499 (19,78%)	31.385 (19,41%)
55-64	42.180 (16,2%)	26.024 (16,09%)
>65	25.548 (9,81%)	14.89,71%)

**Table 4:** Distribution of confirmed culture-positive TB cases registered and treated by gender in 2019 and 2020

Gender	No. (%) of TB cases 2019	No. (%) of TB cases 2020
Male	140,877 (60.57%)	157,764 (60.41%)
Female	91,685 (39.43%)	103,349 (39.58%)
Total	232,562	261,113

**Table 5:** Distribution of total TB cases registered and treated by gender

Gender	No. (%) of TB cases 2019	No. (%) of TB cases 2020
Male	276.705 (57,89%)	322.954 (57,55%)
Female	201.223 (42,11%)	238.146 (42,45%)

**Table 6:** Recovery rate of lung TB confirmed by bacteriological examination by gender in 2019 and 2020

Gender	No. (%) recovery rate 2019 (%)	No. (%) recovery rate 2020 (%)
Female	101.913 (59,85%)	108.704 (59,77%)
Male	68.266 (40,11%)	73.137 (40,23%)
Total	170.179	181.841

**Table 7:** Comparison of full recovery rate of TB cases by gender in 2019 and 2020.

No	Gender	No. (%) recovery rate 2019 (%)	No. (%) recovery rate 2020
	Male	135.646 (55,63%)	156.904(55,55%)
	Female	108.173 (44,37%)	125.512(44,45%)
	Total	243.819	282.416

Success rate of TB cases

**Table 8:** Distribution of TB cure rates by sex in 2019 and 2020

No	Success Rate of Tuberculosis	2019 (%)	2020 (%)
	Male	135.646 (55,63%)	156.904(55,55%)
	Female	108.173 (44,37%)	125.512(44,45%)
	Total	243.819	282.416

**Table 9:** Distribution of mortality rates during treatment in 2019-2020

No	The number of deaths during TB treatment	2019 (%)	2020 (%)
1	The number of deaths during TB treatment	11.993 (2.20%) 543.874	13.174 (3,48%) 378.936

**Table 10:** Distribution of TB incidence rates per province in 2019 and 2020 per 100,000 population

Province	Incidence rate in 2019	Incidence rate in 2020
Aceh	160,62	124,40
North Sumatra	218,57	144,41
West Sumatra	234,36	108,44
Riau	170,2	129,85
Jambi	154,14	83,55
South Sumatra	255,93	119,26
Bengkulu	186,61	75,70
Lampung	209,06	148,88
Bangka Belitung Island	164,03	113,93
Riau Island	277,08	183,21
Jakarta	**443,7**	249,68
West Java	291,86	182,04
Central Java	177,72	122,87
Yogyakarta	125,69	85,70
East Java	178,06	113,74
Banten	247,04	171,98
Bali	**102,72**	**67,43**
West Nusa Tenggara	153,12	110,89
East Nusa Tenggara	146,37	95,68
West Kalimantan	206,45	133,97
Central Kalimantan	151,72	90,06
South Kalimantan	180,34	81,92
East Kalimantan	233,62	121,46
North Kalimantan	284,68	128,17
North Sulawesi	335,58	192,58
Central Sulawesi	195,19	134,29
South Sulawesi	233,53	138,98
Southeast Sulawesi	164,38	106,90
Gorontalo	352,56	192,85
West Sulawesi	209,38	141,56
Maluku	261,8	106,18
North Maluku	184,75	115,19
West Papua	318,98	162,15
Papua	439,5	**297,92**

## Discussion

TB is one of the communicable diseases and it is still endemic in some tropical countries, including Indonesia. The Global Report on TB in 2020 showed that the incidence of TB in Indonesia was high. The archipelago country with more than 17,000 islands needs to be challenged with the elimination of TB in Indonesia. Indonesia has been listed as one of the 30 countries with the highest global TB burden. The other 29 countries including of Angola, Brazil, Bangladesh, China, Congo, Central African Republic, Democratic Republic of Congo, Ethiopia, Gabon, India, Kenya, Lesotho, Mozambique, Mongolia, Myanmar, Nigeria, Namibia, Pakistan, Papua New Guinea, Philippines, Sierra Leone, South Africa, Thailand, Uganda, United Republic of Tanzania, Vietnam, Democratic Peoples of Korea, and Zambia. Since 1995, there have been an increasing number of TB incidents in Indonesia, which ranks second in the world for this statistic. However, the number of new cases each year rises by 25.40/1,000,000 people, and in 2017 there was a reported treatment rate of 88% (Caren *et al.*, 2022). A Global strategy was adopted by WHO to reduce mortality rate due to TB by reducing TB incidence at 80% by 2030 (WHO, 2019).

TB cases in Indonesia decreased in 2020 with a total number of 351,936 compared to 2019 which was 543,874 cases. Also, there was lower CNR compared to 2019. However, treatment increased to 44%. TB cases were also more common in men compared to women, as evidenced from 2019 to 2020. Several factors were found and analyzed to be responsible for the observed decrease in the incidence of TB. Some of the problems have to do with overcoming the problems of delays in diagnosis and non-adherence to treatment. Efforts made for TB elimination in Indonesia have worsened since the COVID-19 pandemic (Caren *et al.*, 2022). Both COVID-19 and TB have an impact on the respiratory system, especially the lungs, such as coughing, fever, and difficulty breathing. COVID-19 and TB have similarities in terms of transmission, through airborne, but COVID-19 transmission is faster and more difficult to control than TB (Revita *et al.*, 2022).. In addition to having a direct impact on health, COVID-19 also has secondary effects such as lockdowns, economic turmoil, disease and reduction of health workers, overwhelmed health facilities (Khan *et al.*, 2021). According to WHO in the Global TB report 2021, it states that case notifications have decreased dramatically due to disruptions in TB services (WHO, 2020). Furthermore, WHO also estimates that nearly 10 million people will suffer from TB in 2020. However, only 5.8 million cases are diagnosed and reported which represents a decrease of 18% from 2018. This decline occurred mainly in Asian countries, especially India, Indonesia, Philippines and China (Rodrigues *et al.*, 2022). The Global TB Network evaluated attendance at TB service units in 33 centres from 16 countries in comparison to the number of TB-related health service activities in 4 months during the COVID-19 pandemic in 2019 and reported that most centres experienced a decline and reduction in new active TB case findings, total number of outpatient active TB visits and newly discovered latent TB cases (Visca *et al.*, 2021). This happened during the lockdown in the first 4 months at the beginning of 2020. Since the pandemic, it has been given priority for COVID-19 in several TB care centres that were first in contact with TB service providers. In addition, patients’ worries about COVID-19 exposure in the community and access issues during the lockdown are linked to the decline in visits to TB clinics (Visca *et al.*, 2021)

Our study showed that in 2020, the majority of TB cases among 45-54 age group stood at a total of 60,785 cases. Same finding was also demonstrated during 2019 with most of the TB cases affecting individuals who were in the 45-54 age group. However, based on the data obtained, TB also occurs in all age groups from 0 year to >65 years, especially in the age group >65 years, it can cause death due to COVID-TB, in accordance with previous research reports that the mortality rate due to COVID-19 increases exponentially due to age factors (Daniel *et al.*, 2022; Zhou *et al.*, 2020). Research conducted by Kristini and Hamidah in 2020 on the potential for transmission of pulmonary TB to family members of patients in Semarang, Central Java, Indonesia, illustrates that pulmonary TB is one of the most common diseases affecting productive age (15-49 years). Patients with TB positive-smear can transmit TB to all age groups. In 2017 in the city of Semarang there were TB sufferers of all types, in the infant and child age group 24%, in the 15–44-year age group 40%, and in the over 55-year age group, it was 22%. The percentage of pulmonary TB of all types in men is greater than in women because men pay less attention to maintaining their own health and men often have contact with risk factors compared to women (Kristini and Hamidah., 2020).

TB is an infectious disease that requires appropriate action and examination. Measures taken in diagnosing TB through bacteriological examination include direct microscopic sputum examination, TB molecular rapid test examination, and culture examination (WHO, 2018). For those who require TB prevention and treatment, every precaution must be taken to guarantee continuity of care. Health authorities must continue to fund TB services even in times of crises like COVID-19. Along with the COVID-19 approach, TB prevention, diagnosis, treatment, and care services focused on the community must be provided (WHO, 2020). In order to treat COVID-19 and TB, accurate diagnostic testing is crucial. People with respiratory symptoms, which may be identical for the two diseases, should have access to tests for both ailments, which are separate from each other. People who are assessed for COVID-19 and TB require generally distinct specimens because the diagnostic processes for these two illnesses are very different. Sputum and numerous other biological specimens can be utilized to detect TB using molecular or culture methods (WHO, 2019^b^). The most frequent method of testing for COVID-19 in outpatients is nasopharyngeal or oropharyngeal swab or wash; however, in patients with severe respiratory disease, sputum, endotracheal aspiration, or bronchoalveolar rinses may be employed. Molecular testing is currently advised for detecting TB-like illnesses and COVID-19 (WHO, 2020).

The number of bacteriologically confirmed, registered, and treated pulmonary TB cases have increased compared to 2019, as many as 232,52 increasing to 261,113. In addition, those who were registered and had been treated increased by 477,928, increasing to 561,100. This percentage is proportional to the increase in the TB cure rate from 2019 to 2020 from 170,179 in 2019 to 181,841 in 2020. The percentage of TB treatment success rate is an indicator that gives an overview of the quality of TB treatment, i.e. how much success is recorded in the treatment of the TB patients who have been treated and reported. This figure describes the number of TB patients who were successful in their treatment, both in the cured category and in the complete treatment category. TB incidence is the P2TB program’s performance indicator in the National Medium-Term Development Plan (RPJMN). TB incidence is an indicator that provides an overview of the burden of TB disease and can provide an indication of how many new TB cases appear each year. The indicator of the percentage coverage of TB treatment success in 2020 reaching the target is an analysis of treatment success in 2020 according to the Directorate General of P2P of the Ministry of Health of the Republic of Indonesia. These data are still temporary data and are subject to change after all Provincial Health Offices submit reports. The achievement of the target was due to various expansions that have been carried out, such as the expansion of TB examination laboratories, expansion of TB RO service facilities so as to support the increasing number of TB cases found and treated, the role of supervisors for swallowing drugs and health care facilities that are getting better, as well as the implementation of mopping up/case sweeping to hospitals in the Province and Regency/City (Indonesian Ministry of Health, 2020).

The success of TB patients’ treatments will determine if the disease is eradicated. In an earlier study, it was reported that several districts in East Java were found to have TSR (Treatment Success Rate) values less than the target, that is <90%. To finish treatment, 20 areas with TSRs under 90% require the deployment of public health promotion and improvement of knowledge of the TB patients (Utomo *et al.*, 2022) This study also describes an increase in TB deaths during treatment from 2019-2020. In October 2021, WHO predicts an increase in deaths from TB for the first time in ten years, the COVID-19 pandemic is to blame. WHO also stated that the decline in TB notifications was caused by reduced access to health services, and caused TB deaths to increase during the pandemic. In addition, if the notification is lower, it also describes a decrease in the transmission of *Mycobacterium tuberculosis*. Furthermore, despite the restrictions on activities due to the pandemic, it does not rule out the possibility of transmission in the household. In addition, there is evidence that pandemics lead to a reduction in the incidence of respiratory illnesses such as influenza. This can illustrate that actions and health services at that time were focused on the COVID-19 pandemic through policy and behavior changes. Which of course also has an impact on TB cases (Dowdy *et al.*, 2022). This study also reports incident rate data for 2019 and 2020. The highest incidence in 2019 was in Jakarta and in 2020 it was in Papua. This shows that the incidence of TB needs to be managed properly. Meanwhile, a reduction in the incidence and mortality due to TB by 20% and 35% is proposed by the WHO in 2020 (Motta *et al.*, 2020). The focus of government’s unit that handles infectious diseases was torn between two, in managing global COVID-19 pandemic, and also other infectious diseases, one of it was Tuberculosis. This could lead to the decrease in the case detection rate, resulting in the declining numbers of new TB cases in 2020.

## Conclusion

There was a decrease in TB cases before the COVID-19 pandemic (2019) compared to during the COVID-19 pandemic (2020).

### Funding:

The present study was supported by a grant from the Penelitian Unggulan Fakultas Scheme, Faculty of Medicine, Universitas Airlangga (grant no. 66/UN3.15/PT/2022)

**List of Abbreviations:**
COVID-19: Coronavirus Disease 2019;
SARS-CoV-2: Severe Acute Respiratory Syndrome caused by Corona Virus 2;
TB: Tuberculosis;
WHO: World Health Organization
